# The prognostic role of Geriatric 8 in patients with cancer: a meta-analysis and systematic review

**DOI:** 10.1093/oncolo/oyaf118

**Published:** 2025-06-21

**Authors:** Runzhi Chen, Dongmei Yang, Mengxing Tian, Huiting Xu, Xin Jin

**Affiliations:** Department of Medical Oncology, Hubei Cancer Hospital, Tongji Medical College, Huazhong University of Science and Technology, Wuhan, Hubei 43000, China; Department of Medical Oncology, Hubei Cancer Hospital, Tongji Medical College, Huazhong University of Science and Technology, Wuhan, Hubei 43000, China; Department of Clinical Nutrition, Hubei Cancer Hospital, Tongji Medical College, Huazhong University of Science and Technology, Wuhan, Hubei 43000, China; Department of Medical Oncology, Hubei Cancer Hospital, Tongji Medical College, Huazhong University of Science and Technology, Wuhan, Hubei 43000, China; Department of Clinical Nutrition, Hubei Cancer Hospital, Tongji Medical College, Huazhong University of Science and Technology, Wuhan, Hubei 43000, China

**Keywords:** Geriatric 8, frailty, cancer, overall survival, meta-analysis

## Abstract

**Objective:**

Previous studies have reported conflicting results regarding the association between the Geriatric 8 (G-8) geriatric screening tool and prognosis in patients with cancer. This meta-analysis aimed to evaluate the prognostic value of the G-8 score in patients with cancer.

**Methods:**

PubMed, Cochrane Library, Embase, and Medline databases were searched to identify trials exploring the association between G-8 score and prognosis in patients with cancer. Meta-analyses of overall survival (OS) and progression-free survival (PFS) between the high and low G-8 scores were performed. The quality of the included studies was assessed using the Quality In Prognosis Studies tool.

**Results:**

A total of 42 studies involving 9053 patients with cancer were included. The prevalence of frailty, evaluated using the G-8 tool across trials, ranged from 27% to 91%. A low G-8 score was associated with poor OS (Hazard ratio [HR] 2.11; 95% CI:1.93-2.31, *P* <.001) and PFS (HR 1.78, 95% CI,1.55-2.05, *P* <.001) in patients with cancer. Overall survival were shorter in patients with low G-8 scores than in those with high G-8 scores in digestive system tumors, head and neck cancer, lung cancer, gynecologic tumors, hematologic malignancies, and prostate cancer. The predictive role of the G-8 tool was also confirmed in subgroups with G-8 cutoff values of 9-14. Patients with low G-8 scores had more advanced disease stages and higher ECOG performance status scores.

**Conclusions:**

The prevalence of frailty was high among patients with cancer according to the G-8 geriatric screening tool. Decreased G-8 scores are significantly associated with poor survival in patients with cancer. G-8 is a promising tool for frailty screening.

**Implications for Practice**

The Geriatric 8 (G-8) screening tool is effective for screening frailty in patients with cancer and is capable of predicting poor overall and progression-free survival. Its simplicity and prognostic value across diverse cancer types makes it a practical tool to identify frailty. Clinicians should integrate G-8 into routine assessments to identify patients who require Comprehensive Geriatric Assessment. In addition to identifying frail patients, G-8 screening in oncologic assessment provides a more comprehensive understanding of patients' health status, guides treatment decisions, and promotes patient-centered care, and also contributes to improved resource allocation.

## Introduction

Cancer is the second leading cause of mortality worldwide, with an estimated 16.8% of all deaths attributed to the disease.^1^ In 2022, the incidence of cancer reached nearly 20 million new cases globally, resulting in ~9.7 million cancer-related fatalities.^2^ It was estimated that 4.8 million new cancer cases and 2.5 million deaths were in China.^3^ The incidence of cancer has increased steeply among the elderly population. Approximately 30% of newly diagnosed cancer patients in the United States fall within the 65-74 years age bracket. Another 25% were 75 years or older.^4^ As age advances, the efficacy of antitumor treatments tends to diminish and tolerance to therapies is significantly compromised in elderly patients with cancer.^5^ Thus, they are more vulnerable during the treatment process, and antitumor treatment presents greater challenges.^6^

Frailty is an age-related disorder that is a significant cause of poor prognosis and challenging to manage in elderly patients with cancer.^7^ Frailty involves multiple interrelated physiological systems, including the brain and endocrine, immune, skeletal muscle, respiratory, cardiovascular, renal, hematopoietic, and coagulation systems.^8^ Common clinical presentations of frailty include extreme fatigue, unexplained weight loss, frequent infections, balance and gait impairment, delirium, and fluctuating disabilities. Previous research indicated that the prevalence of frailty among community-dwelling individuals aged over 65 years was as high as 59.1%.^9^ Studies have shown that patients with cancer are particularly susceptible to frailty.^10^ A systematic review revealed that the prevalence of frailty in older patients with cancer is high, with a median estimate of 43%.^11^ On the one hand, the incidence of frailty in elderly cancer patients is also higher.^12^ Frailty, in turn, increases the risk of developing various diseases, including cancer.^13^ However, anticancer treatments can also cause frailty.^14^ In addition, in cancer patients receiving anticancer treatment, frailty was a significant predictor of clinical outcomes including a high incidence of postoperative complications, poor tolerance to chemotherapy, high risk of tumor recurrence, and short survival time.^15^ Moreover, a prospective study demonstrated a positive correlation between frailty and the risk of developing cardiovascular diseases and type 2 diabetes mellitus among long-term cancer survivors, even in its early stages.^16^ This evidence highlights the importance of frailty assessment in cancer patients to improve their long-term prognosis.

Some guidelines recommend using the Comprehensive Geriatric Assessment (CGA) to assess frailty in elderly patients with cancer.^17^ Studies have demonstrated that CGA is recognized as a process and the accepted gold standard method for the better identification and management of frail older patients.^18-20^ The CGA is a multidimensional, interdisciplinary diagnostic process that specifically and comprehensively assesses functional and cognitive abilities, social support, economic status, environmental factors, and physical and mental health.^21^ However, the cost and complexity of CGA limit its use in clinical practice.^22^ Thus, most recommendations from international and national medical societies agree that frailty assessment in older cancer patients should begin with rapid screening to identify patients who may be vulnerable and could benefit from CGA.^23,24^ Some tools have been developed to screen for frailty, such as the Vulnerable Elders Survey-13 (VES-13), Triage Risk Screening Tool (TRST), and Geriatric 8 (G-8) geriatric screening tool.^25,26^ In addition to identifying frail patients, frailty screening in oncologic assessment provides a more comprehensive understanding of patients’ health status, guides treatment decisions, and promotes patient-centered care while also facilitating CGA and improving resource allocation. A previous systematic review exploring the diagnostic accuracy of various screening tools indicated that G-8 and VES-13 were the most commonly studied tools.^27^ The G-8 tool, which as a geriatric screening tool is easily obtained, has been verified as a promising tool for frailty in cancer.^26^ The components of the G-8 tool include questions related to nutritional status, weight loss, body mass index, motor skills, psychological status, number of medications, self-perception of health, and age (<80, 80-85, and >85 years).^26^ The assessment takes 3-5 minutes to complete, with a total score of 0-17. A score of 14 or less indicates the presence of a risk profile usually. The G-8 tool provided a good sensitivity estimate (85%) compared with a reference exam consisting of 7 CGA questionnaires from a multicenter prospective study.^26^ In recent years, the G-8 tool has been applied to patients with cancer to predict clinical outcomes, with inconsistent results.^28,29^ Most trials have found that G-8 score is an independent predictor of survival in patients with cancer. However, other studies have shown no significant differences in overall survival (OS) and progression-free survival (PFS) between low and high G-8 scores in elderly patients with cancer.^30,31^

Several published systematic reviews have explored the impact of G-8 scores on the clinical outcomes of patients with cancer. Two of these studies did not perform a meta-analysis.^32,33^ Another systematic review focused on the screening accuracy of G-8 tool and did not analyze G-8 tool’s prognostic predictive effect.^27^ One published meta-analysis reviewed the effect of the G-8 tool on the prognosis of patients with cancer; however, it was restricted to patients undergoing surgical treatment. This study found that the G-8 tool was effective and efficient in evaluating the risk of postoperative complications in only 11 published studies.^34^ Another meta-analysis that explored the relationship between G-8 and OS included 8 trials. Moreover, no subgroup analyses were performed in this meta-analysis.^35^ Many recently published studies have investigated the predictive role of the G-8 score for survival time in patients with cancer. Therefore, we conducted a meta-analysis of these studies to evaluate the prognostic value of the G-8 tool in cancer patients. In addition, we conducted subgroup analyses to analyze the prognostic effects of different G-8 cutoff values and the prognostic performance of G-8 in specific cancer types. We also investigated the correlation between a low G-8 score and clinical characteristics such as disease stages and ECOG performance status.

## Methods

This meta-analysis was conducted in accordance with the Preferred Reporting Items for Meta-Analyses statement. We searched all published articles in the PubMed, Embase, Medline, and Cochrane Library databases until January 25, 2025, for all references using the keywords “G-8” and “cancer” and other related words. The complete search used for PubMed was (geriatric 8 [Title/Abstract] OR “geriatric‑8” [Title/Abstract] OR “G8” [Title/Abstract] OR “G-8” [Title/Abstract] OR “Vulnerable Elders Survey-13” [Title/Abstract] OR “VES-13” [Title/Abstract]) AND (“cancer” [Title/Abstract] OR “tumor” [Title/Abstract] OR “malignancy” [Title/Abstract]). The inclusion criteria were as follows: (1) the study was designed as a prospective cohort or retrospective study, (2) patients were diagnosed with cancer, (3) patients had G-8 scores calculated before treatment, and (4) outcomes included PFS or OS. Conference abstracts, case reports, presentations, and unpublished trial data were excluded. Studies in which participants had no G-8 scores were also excluded.

### Study selection

Two researchers independently examined the title and abstract of each article. After excluding the articles that did not meet the inclusion criteria, the full texts of the remaining articles were reviewed. When opinions differed on whether an article was excluded, a final conclusion was reached by consulting a third reviewer.

### Data extraction

Two researchers independently extracted data from the included articles and checked the extracted data against each other. Data extracted from the articles included the year of the study, name of the first author, characteristics of the study participants (country, age, type of cancer, and stage), G-8 score, cutoff values of the G-8 score, antitumor therapy method, and outcomes (PFS and OS).

### Quality assessment

The Quality In Prognosis Studies (QUIPS) tool was used to evaluate the risk of bias in the included studies. Two researchers evaluated the quality of studies included in this meta-analysis. This tool includes 6 aspects: study population, study loss to follow-up, prognostic factor measurement, outcome measurement, confounding factors, and statistical analysis and reporting. In accordance with established criteria, studies exhibiting a low risk of bias were determined based on the presence of 5 or more of the 6 criteria with low risk of bias. Conversely, if 2 or more criteria indicated a high risk of bias, the study was considered as having a high risk of bias. Otherwise, the studies were considered to have a moderate risk of bias.

### Data synthesis and statistical analysis

All meta-analyses were conducted using Stata 14.0. Cochran’s Q statistic was used to detect between-study heterogeneity. Heterogeneity was quantified by *I*^2^. If *I*^2^ was greater than 50%, the studies were considered heterogeneous, and a random-effects model was used. If *I*^2^ was less than or equal to 50%, the heterogeneity among studies was considered low, and a fixed-effects model was used for analysis. Pooled hazard ratios (HR) and the corresponding 95% CIs were calculated for OS and PFS. Most HRs were extracted from the Cox regression analysis in this study. If both univariate and multivariate Cox regression analyses were available, HRs from the multivariate regression were extracted for calculation. If the article did not provide HRs, they were extracted from the Kaplan–Meier survival curves. Funnel plots and Egger’s test were used to identify publication bias. Predefined subgroups including design of the studies, the country in which the clinical study was conducted, number of patients, type of cancer, G-8 tool cutoff values, and type of therapy were also conducted to further explore the effect of the G-8 score on cancer prognosis. A sensitivity analysis was performed using the leave-one-out evaluation procedure.

## Results

### Selection of literature

**Figure 1.**
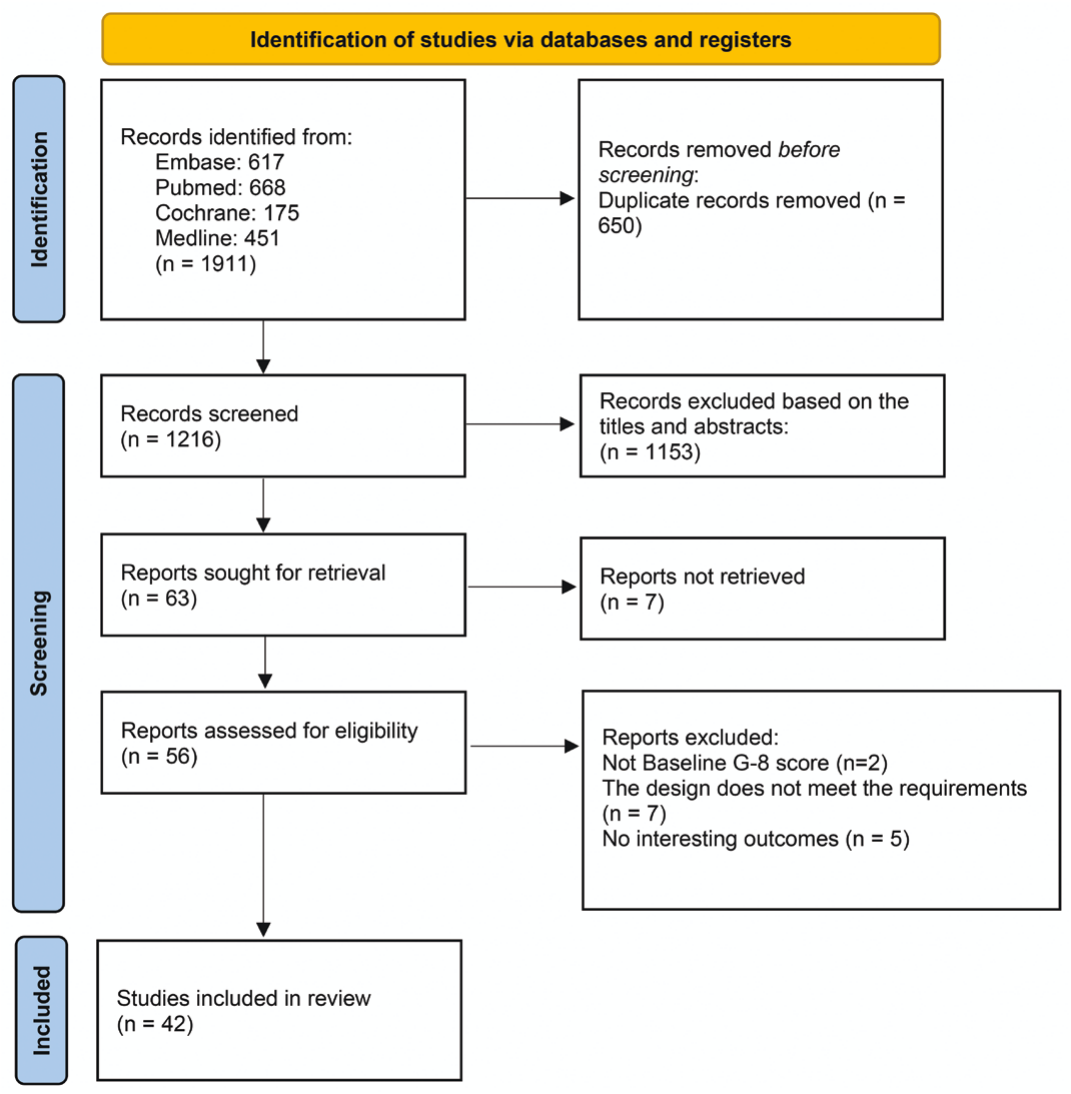
A flow chart of the study selection process.

In total, 1911 records were obtained from the 4 databases. After removing duplicate articles (*n* = 650) using the Endnote software, 1216 studies remained. Two independent researchers (X. Jin and R.Z. Chen) read the titles and abstracts of these articles and initially excluded 1153 studies. We screened the full texts of the remaining 63 articles and excluded 14 studies for several reasons. Finally, 42 studies were deemed eligible and subsequently included in the meta-analysis (Figure 1).

### Patient characteristics

Among the 42 studies,^15,28-31,36-72^ the sample sizes ranged from 26 to 1333, and 23 studies had fewer than 100 people. A total of 9053 patients were included in the meta-analysis. All studies utilized the G-8 screening tool to evaluate frailty, and the cutoff value used to define frailty ranged from 9 to 14. Nine of the 42 studies used 10 as the cutoff value,^30,39,43,48-51,57,67^ and 28 used 14 as the cutoff value.^15,28,31,36-38,40-42,44-46,54-56,59-66,68-72^ Three studies used 12^47, 52, 63^, 1 used 13,^58^ and 4 used 11^15, 53, 62, 68^ as the cutoff value for G-8. The prevalence of frailty across the trials ranged from 27% to 91% (median prevalence 65.4%). The mean or median ages in the included studies ranged from 62 to 85 years. Of the included studies, 19 were retrospective studies,^28,30,31,37,38,42,43,46,47,50,52-55,57,58,66,70,72^ 22 were prospective studies,^15,29,36,39,41,44,45,48,49,51,56,59-65,68,69,71^ and 1 was a case-control study.^40^ Twenty-three studies were conducted in Europe and 19 in Asia. Four studies involved patients with head and neck cancer, 7 with lung cancer, 11 with digestive system tumors, 4 with prostate cancer, and 2 with gynecologic tumors (Table 1).

**Table 1.** Characteristics of the trials included in the meta-analysis.

Study	Year	Country	Study design	*n*	Age	Population	Tumor stage	Therapy	Cut-off value	Prevalence of frailty	Outcomes
Lies Pottel	2015	Belgium	PS	100	72 (65-86)	Head and neck cancer	Mixed	Radiotherapy	≤14	68%	OS, Stage
Masahiro Takahashi	2017	Japan	RS	264	75 (70-91)	Elderly cancer patients	Mixed	NR	≤14	83%	OS, ECOG PS
Yoko Agemi	2019	Japan	PS	76	79 (70–95)	Lung cancer	Mixed	Mixed	≤14	76%	OS, ECOG PS
Elise Deluche	2019	France	RS	89	74 (65-87)	Glioblastoma	NR	Mixed	≤14	81%	OS, ECOG PS
Toshio Kubo	2020	Japan	RS	95	79 (75–90)	Non-small cell lung cancer	Advanced	Immunotherapy	≤11	NR	OS
Masaki Momota	2020	Japan	PS	96	73 (68-77)	Metastatic prostate cancer	Advanced	Mixed	≤14	70%	OS
Kristian Kirkelund Bentsen	2021	Denmark	PS	46	72 (52-87)	Non-small cell lung cancer	Localized	Stereotactic body radiotherapy	≤14	72%	OS, ECOG PS
Anne-Laure Couderc	2020	France	PS	228	78.7 (67-95)	Lung cancer and thoracic tumors	Mixed	Mixed	≤14	91%	OS
Katharina Anic	2023	Germany	RS	150	71	Endometrial cancer	Mixed	Surgery	≤14	39%	OS, PFS, Stage
Li-Yuan Bai	2022	China	PS	49	76 (70-87)	Pancreatic cancer	advanced	Chemotherapy	≤10	47%	OS, PFS
Ajay T. Bakas	2023	Netherlands	case‐control	135	62 (58-67)	Head and Neck cancer	Mixed	Mixed	≤14	/	OS, Stage
Giuseppe Luigi Banna	2022	Italy	PS	234	78 (73-82)	Prostate cancer	advanced	Endocrine therapy	≤14	62%	OS, PFS
Carlotta Becherini	2023	Italy	RS	26	76 (70-88)	Head and Neck cancer	advanced	Immunotherapy	≤10	58%	OS, PFS
Kazuma Kobayshi	2022	Japan	PS	46	77.5 (71-87)	Gastric cancer	Advanced	Chemotherapy	≤10	67%	OS, PFS
Ryo Ishii	2021	Japan	PS	78	79 (70–97)	Head and neck cancer	Mixed	Mixed	≤10	29%	OS
Eva Jespersen	2021	Denmark	PS	170	75.5 (72-79)	Gastrointestinal cancer	Advanced	Chemotherapy	≤14/≤11	74%	OS
Shin Lee	2021	Japan	RS	388	77 (65-96)	Diffuse large B-cell lymphoma	NR	Chemotherapy	≤14/≤9	85%	OS, ECOG PS, Stage
Mauro Loi	2021	Italy	RS	42	85 (80-91)	Hepatocellular carcinoma	NR	Stereotactic Body Radiotherapy	≤10	62%	OS
Ellen R. M. Scheepers	2020	Netherlands	RS	177	79.6 (70.4-96.7)	Breast cancer	Localized	Mixed	≤14	79%	OS
Pastora Beardo	2019	Spain	RS	70	≥75	Prostate cancer	Advanced	Endocrine therapy	≤14	NR	OS
Kei Fujita	2023	Japan	RS	100	75 (60-90)	Acute myeloid leukemia	NR	Mixed	≤12	56%	OS
Maria Gavriatopoulou	2019	Greece	PS	110	83 (80-92)	Octogenarian myeloma	NR	Chemotherapy	≤10	27%	OS
Kenji Morimoto	2023	Japan	PS	44	72.5 (69-77.5)	Small cell lung cancer	Advanced	Immunotherapy + Chemotherapy	≤11	52%	OS, PFS, EGCO PS
Makoto Kadokura	2022	Japan	RS	77	72 (65-91)	Pancreatic cancer	Advanced	Chemotherapy	≤10	39%	OS
Harald Krenzlin	2021	Germany	RS	104	76.6 (70-89)	Glioblastoma	NR	Mixed	≤12	65%	OS
Zhaohui Liao	2023	China	RS	164	73 (70-85)	Nasopharyngeal carcinoma	Mixed	Radiotherapy	≤14	39%	OS, PFS
Gabor Liposits	2023	Denmark	PS	154	78 (75-81)	Colorectal cancer	Advanced	Chemotherapy	≤14	84%	OS, PFS
Ina Valeria Zurlo	2022	Italy	RS	90	78 (75-87)	Gastric and Gastro-Esophageal Cance	Advanced	Chemotherapy	≤14	73%	OS, PFS, EGCO PS
Michał Wilk	2022	Poland	PS	49	71 (67-79)	Prostate cancer	Advanced	Endocrine therapy	≤14	47%	OS, PFS
Masanobu Takahashi	2021	Japan	PS	30	73 (65-81)	Colorectal cancer	Advanced	Chemotherapy	≤14	73%	OS, PFS
Shinsuke Shiotsu	2022	Japan	PS	33	78.5 (67.0-87.0)	Non-small cell lung cancer	Advanced	Immunotherapy	≤10	55%	OS, PFS
Minit Shah	2022	India	RS	308	71 (66-74)	Malignant tumor	Mixed	Mixed	≤14/≤11	84%	OS, EGCO PS
Shuhei Sekiguchi	2022	Japan	RS	101	80 (75-98)	Hepatocellular carcinoma	Advanced	Targeted therapy	≤10	32%	OS, PFS
Hánah N. Rier	2022	Netherlands	PS	291	72 (68-77)	Malignant tumor	NR	Chemotherapy	≤14	63%	OS
Jessica Pearce	2022	UK	RS	514	76.0 (51.0-96.0)	Gastro-esophageal cancer	Advanced	Chemotherapy	≤14	89%	OS, PFS
Junichi Nakazawa	2021	Japan	PS	93	76 (70-88)	Gastrointestinal cancer	Advanced	Chemotherapy	≤14/≤12	82%	PFS
Katharina Anic	2022	Germany	RS	110	70.88	Ovarian cancer	Mixed	Mixed	≤14	49%	OS, PFS
Toshiya Maebayashi	2018	Japan	RS	43	78 (65-89)	Early stage Non-small cell lung cancer	Localized	Stereotactic body radiotherapy	≤13	NR	OS
Claudia Martinez-Tapia	2017	French	PS	1333	80 (76-84)	Elderly cancer patients	Mixed	Mixed	≤14	84%	OS
J.G. Middelburg	2020	Netherlands	PS	402	72 (65-96)	Elderly cancer patients	NR	Radiotherapy	≤14	29%	OS
Abhijith R. Rao	2024	India	PS	1175	68 (64-72)	Malignant tumor	Mixed	Mixed	≤14	86%	OS
Pierre Soubeyran	2014	France	PS	1167	78 (70-98)	Elderly cancer patients	Mixed	Mixed	≤14	68%	OS

Abbreviations: RS, retrospective study; PS, prospective study; OS, overall survival; PFS, progress-free survival; EGCO PS, ECOG performance status; NR, not reported.

### Risk of bias of included studies

Twenty-four of the included studies were evaluated to have an overall low risk of bias. Sixteen studies had a moderate risk of bias and 2 studies had a high risk of bias according to the QUIPS tool. The detailed bias results for each article are shown in the supplementary files (Table S1).

### Association of G-8 score with OS

**Figure 2.**
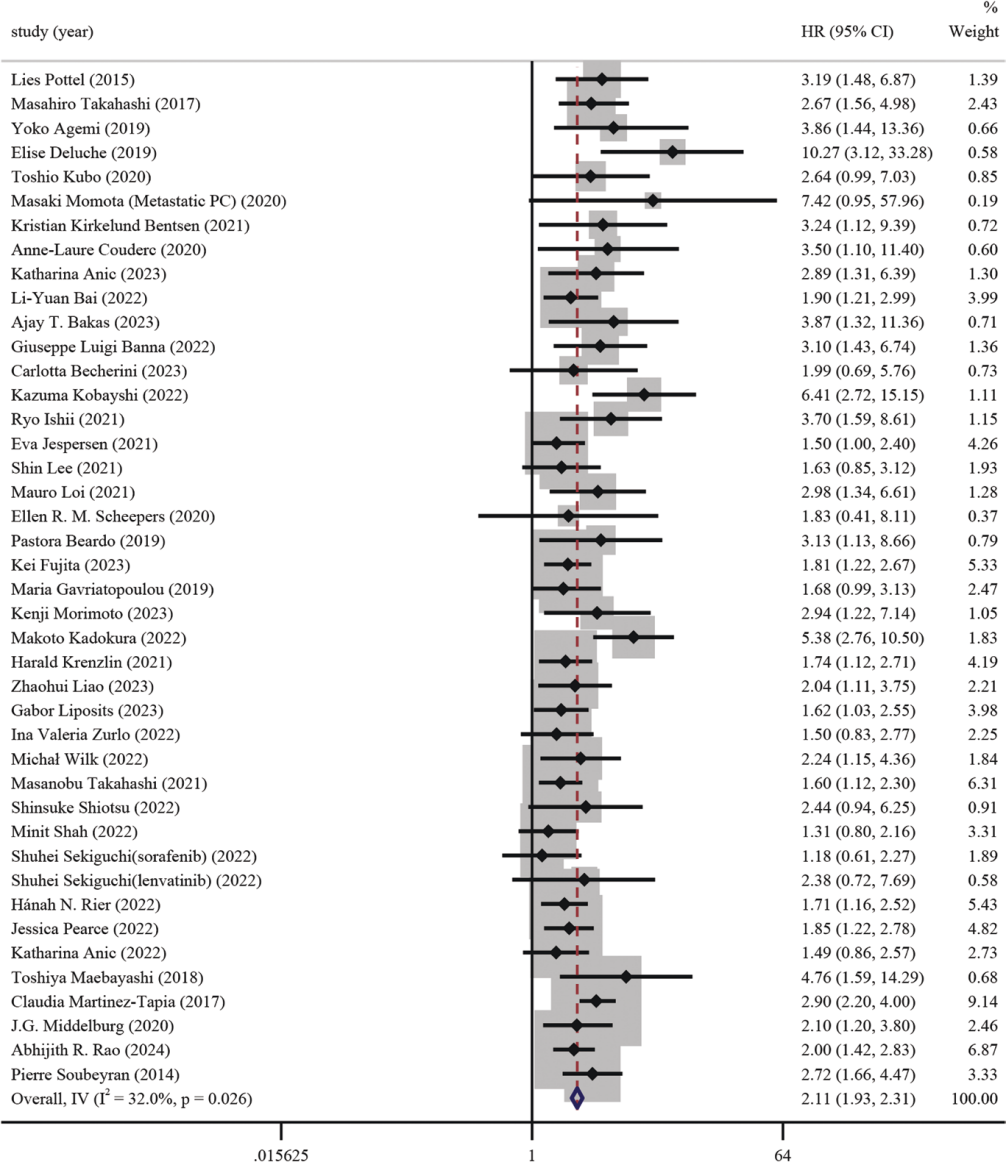
The forest plot for the association between G-8 score and overall survival.

Forty-one studies investigated the relationship between frailty based on the G-8 and OS in patients with cancer. The results of this meta-analysis indicate that frailty evaluated using the G-8 tool is a strong predictor in patients with cancer. Because low heterogeneity was detected among the trials (*I*^2^ = 32.0%), a fixed-effects model was used. The association of frailty and shorter OS was observed with a pooled HR of 2.11 (95% CI,1.93-2.31, *P* <.001) (Figure 2).

### Association of G-8 score with PFS

**Figure 3.**
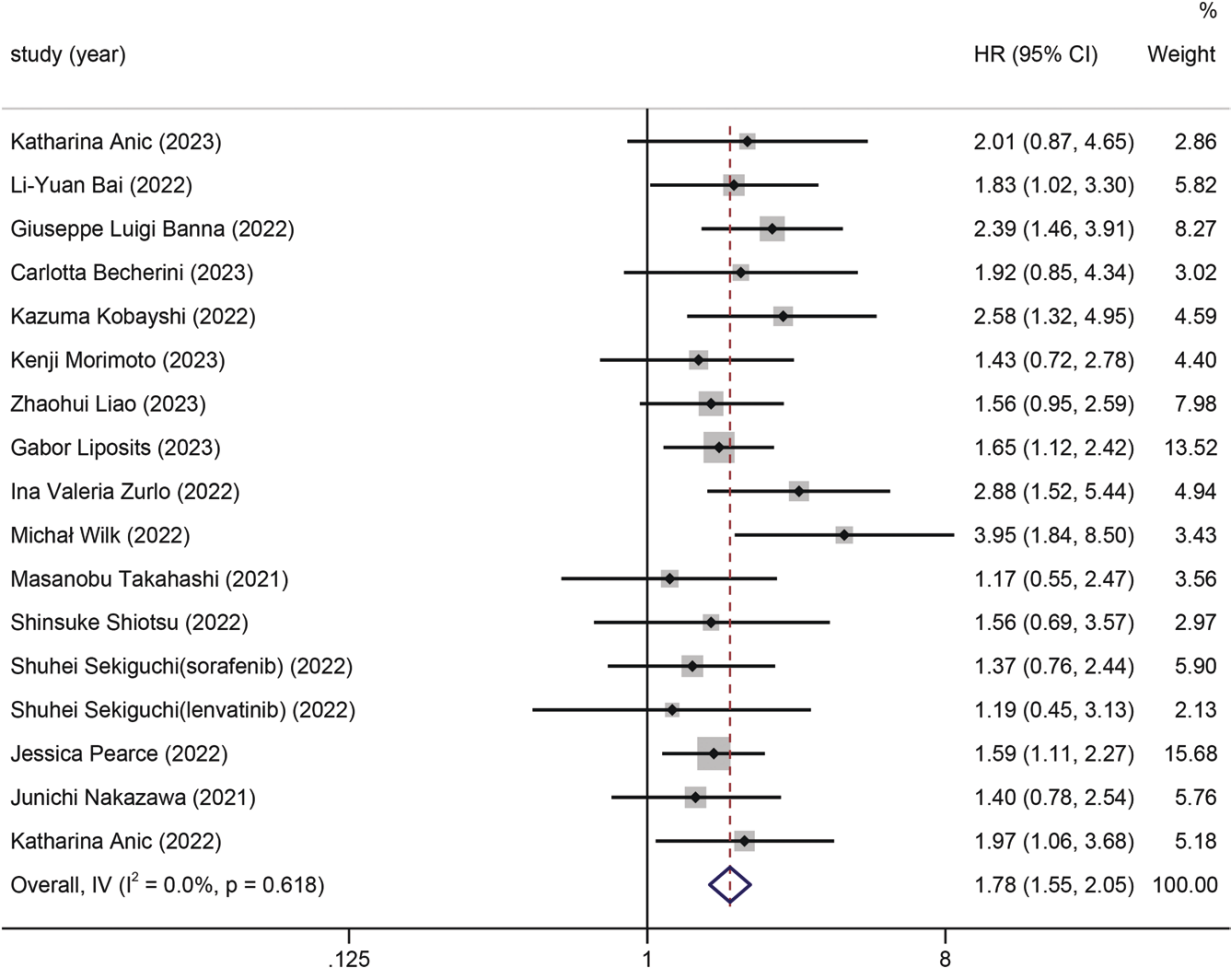
The forest plot for the association between G-8 score and progression-free survival.

A total of 16 articles with 1889 patients reported a relationship between G-8 score and PFS. Because a low heterogeneity (*I*^2^ = 0%, *P* =.618) was detected, a fixed-effects model was used for the meta-analysis. The results demonstrated that high G-8 scores were associated with increased PFS in patients with cancer (HR 1.78, 95% CI, 1.55-2.05, *P* <.001) (Figure 3).

The correlation between a low G-8 score and disease stages/ECOG performance status.

**Figure 4.**
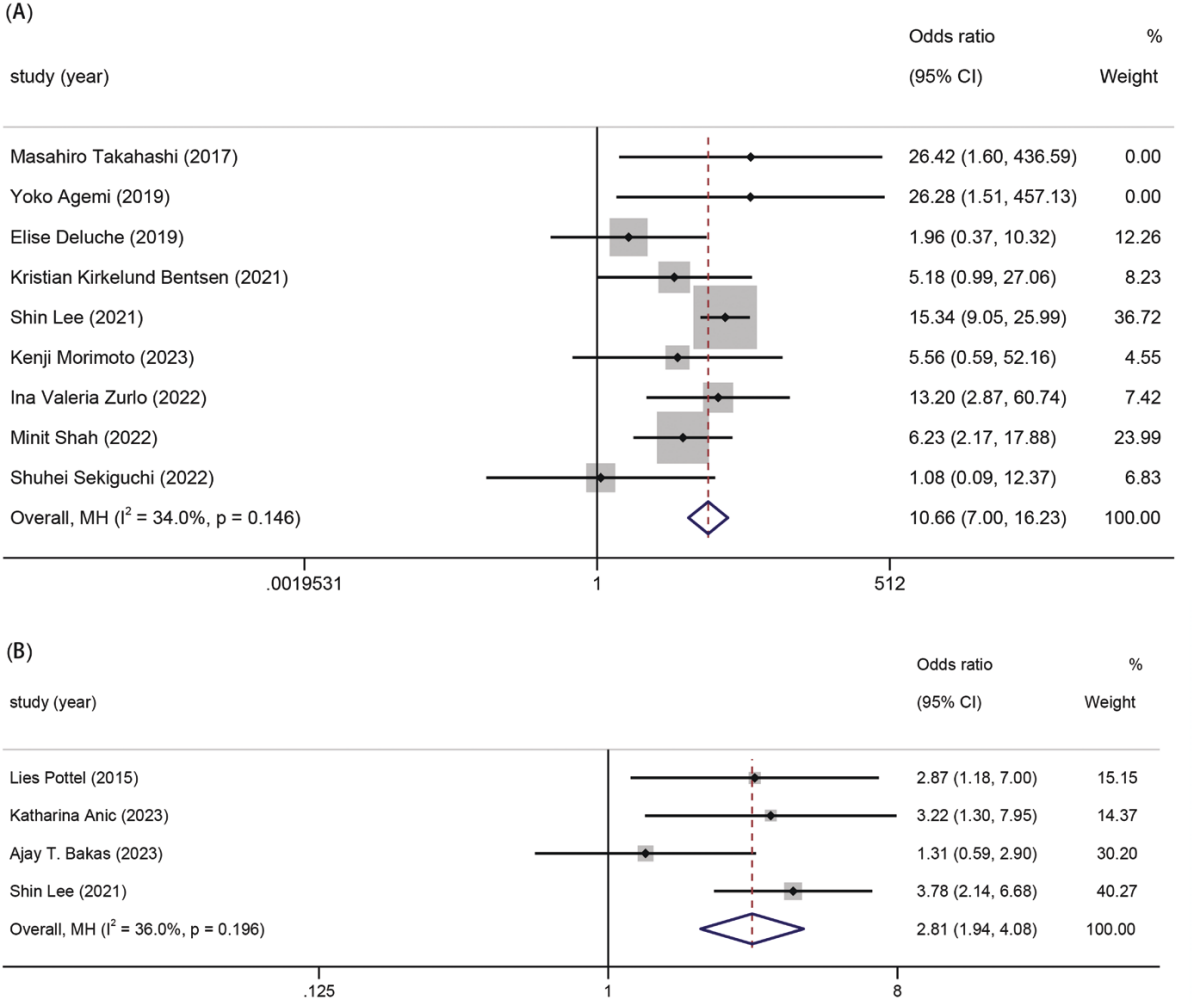
The forest plots for the association between G-8 score and disease stages/ECOG performance status (A) ECOG performance status and (B) stages.

We extracted data from the included studies to explore the correlation between low G-8 scores and stages/ECOG performance status. The results showed that patients with frailty based on G-8 had more advanced disease stages (III–IV vs I–II, OR 2.81, 95% CI,1.94-4.08, *P* <.001). Furthermore, the results demonstrated that patients with low G-8 sores had high PS scores (2/3/4 vs 0/1, OR 10.66, 95% CI,7.00-16.23, *P* <.001) (Figure 4A and B).

### Subgroup analysis of G-8 score in cancer

In most subgroups, OS was lower in patients with low G-8 scores than in those with high G-8 scores. The results showed that decreased G-8 scores were significantly associated with poor OS regardless of the study country, study design, sample size >100, or age >75 years. As presented in Table 2, stratification by G-8 score cutoff revealed an HR of 2.07 (95% CI, 1.86-2.30, *P* <.0001, *I*² = 27.0%) for the cutoff value of 14 and an HR of 1.97 (95% CI, 1.55-2.50, *P* <.0001, *I*² = 0.0%) for the cutoff value of 11 for OS. Similar results were observed in PFS subgroup analysis. The results from subgroup analyses showed that high G-8 scores were associated with increased OS (HR 1.97, 95% CI,1.59-2.44, *P* <.001) and PFS (HR 1.73, 95% CI,1.43-2.10, *P* <.001) in patients treated with chemotherapy. For patients treated with immune checkpoint inhibitors, similar significant associations were observed (Table 2). Additionally, based on subgroup analyses, an increased G-8 score was significantly associated with longer OS/PFS in digestive system tumors, head and neck cancer, gynecologic tumors, and prostate cancer. When the cut-off values of the G-8 tool were set as 14 and 11, there was a significant association between G-8 scores and OS in all these 4 types of tumors (Table 3). However, in several subgroups, including the lung cancer subgroup, the cutoff value of 11, and the radiotherapy subgroup, no significant correlation between G-8 scores and FPS was observed. Moreover, G-8 scores were not significantly with OS in patients with glioblastoma (Table 2).

**Table 2.** Subgroup analyses of the effect of G-8 score on survival.

	OS				FPS			
Parameters	*N*	Heterogeneity	HR (95%CI)	*P*	*N*	Heterogeneity	HR (95%CI)	*P*
Total	42 (41)	32.0%	2.11 (1.93, 2.31)	**<.001**	17 (16)	0%	1.78 (1.55 2.05)	**<.001**
**Design**								
PS	21	28.1%	2.02 (1.75, 2.35)	**<.001**	9	12.4%	1.89 (1.54, 2.31)	**<.001**
RS	20 (19)	37.6%	2.00 (1.73, 2.31)	**<.001**	8 (7)	0.0%	1.72 (1.40, 2.11)	**<.001**
Case-control	1	/	3.87 (1.32, 11.35)	**.014**	/			
**Cutoff values**								
≤9	1 (1)	/	1.82 (1.26, 2.64)	**.002**				
≤10	10 (9)	52.7%	2.56 (1.81, 3.62)	**<.001**	6 (5)	0.0%	1.73 (1.30, 2.30)	**<.001**
≤11	4 (4)	0.0%	1.97 (1.55, 2.50)	**<.001**	1	/	1.43 (0.73, 2.81)	.299
≤12	2 (2)	0.0%	1.78 (1.33, 2.39)	**<.001**	1	/	2.02 (1.22, 3.35)	**.007**
≤13	1 (1)	/	4.76 (1.59, 14.27)	**.005**				
≤14	27 (27)	27.0%	2.07 (1.86, 2.30)	**<.001**	10	14.0%	1.82 (1.54, 2.16)	**<.001**
**Country**								
Europe	23	21.6%	2.13 (1.89, 2.41)	**<.001**	8	4.5%	1.97 (1.64, 2.38)	**<.001**
Asia	19 (18)	44.0%	2.09 (1.82, 2.39)	**<.001**	9 (8)	0.0%	1.56 (1.25, 1.93)	**<.001**
**Number of patients**								
≤100	22 (21)	43.3%	2.31 (1.99, 2.69)	**<.001**	11 (10)	12.1%	1.80 (1.46, 2.22)	**<.001**
>100	20	9.7%	2.00 (1.79, 2.25)	**<.001**	6	0.0%	1.76 (1.45, 2.14)	**<.001**
**Age**								
≤75	18	42.7%	2.06 (1.81, 2.35)	**<.001**	6	19.6%	1.79 (1.36, 2.34)	**<.001**
>75	23 (22)	26.2%	2.15 (1.89, 2.43)	**<.001**	11 (10)	0.0%	1.78 (1.51, 2.10)	**<.001**
**Type of cancer**								
Head and neck cancer	5	0.0%	2.70 (1.88, 3.89)	**<.001**	2	0.0%	1.65 (1.10, 2.53)	**.021**
Lung cancer	6	0.0%	3.14 (2.08, 4.73)	**<.001**	2	0.0%	1.48 (0.88, 2.50)	.140
Glioblastoma	2	86.8%	3.87 (0.69, 21.83)	.125				
Prostate cancer	4	0.0%	2.80 (1.80, 4.36)	**<.001**	2	14.6%	2.77 (1.83, 4.19)	**<.001**
Gynecologic tumors	2	45.0%	1.85 (1.18, 2.90)	**.008**	2	0.0%	1.98 (1.20, 3.27)	**.007**
Digestive system tumors	11 (10)	57.4%	2.02 (1.56, 2.62)	**<.001**	9 (8)	0.0%	1.67 (1.40, 2.00)	**<.001**
Hematologic malignancies	3	0.0%	1.74 (1.30, 2.32)	**<.001**				
Other cancers	9	28.6%	2.20 (1.89, 2.57)	**<.001**				
**Type of therapy**								
Chemotherapy	12	50.6%	1.97 (1.59, 2.44)	**<.001**	7	0.0%	1.73 (1.43, 2.10)	**<.001**
Immune checkpoint inhibitor	4	0.0%	2.52 (1.56, 4.08)	**<.001**	3	0.0%	1.60 (1.03, 2.48)	**.037**
Radiotherapy	5	0.0%	2.49 (1.78, 3.48)	**<.001**	1	/	1.56 (0.95, 2.58)	.082
**Stage**								
Advanced stage	18	39.1%	1.99 (1.72, 2.30)	**<.001**				
Localized	3	0.0%	3.34 (1.69, 6.58)	**.001**				

Bold values indicate statistically significant results (p < 0.05). Abbreviations: *N*, number of comparisons (studies); OS, overall survival; PFS, progress-free survival; HR, Hazard Ratio; CI, confidence interval.

**Table 3.** Stratification analysis of the effect of G-8 score on overall survival in specific cancer.

	Digestive system tumors-OS				Head and neck cancer-OS				Lung cancer-OS				Prostate cancer-OS			
Parameters	*N*	Heterogeneity	HR (95%CI)	*P*	*N*	Heterogeneity	HR (95%CI)	*P*	*N*	Heterogeneity	HR (95%CI)	*P*	*N*	Heterogeneity	HR (95%CI)	*P*
**Total**	11 (10)	57.4%	2.02 (1.56, 2.62)	**<.001**	5	0.0%	2.70 (1.88, 3.89)	**<.001**	6	0.0%	3.14 (2.08, 4.73)	**<.001**	4	0.0%	2.80 (1.80, 4.36)	**<.001**
**Design**																
PS	5 (5)	58.9%	1.90 (1.36, 2.65)	**<.001**	2	0.0%	3.41 (1.93, 6.02)	**<.001**	4	0.0%	3.01 (1.84, 4.94)	**<.001**	3	0.0%	2.73 (1.67, 4.46)	**<.001**
RS	6 (5)	61.2%	2.17 (1.40, 3.36)	**.001**	2	0.0%	2.03 (1.20, 3.44)	**.009**	**2**	0.0%	**3.43 (1.65, 7.12)**	**.001**	1	0.0%	3.13 (1.13, 8.66)	**.028**
Case-control	/	/	/	**/**	1	/	3.87 (1.32, 11.35)	**.014**		**/**	**/**	**/**				
**Cutoff values**																
≤10	6 (5)	69.3%	2.79 (1.64, 4.74)	**<.001**	2	0.0%	2.91 (1.50, 5.63)	**.002**	1	/	2.44 (0.95, 6.29)	.065	/	/	/	**/**
≤ 11	1 (1)	/	2.10 (1.46, 3.02)	**<.001**					2	.0%	2.80 (1.45, 5.40)	**.002**	/	/	/	**/**
≤13	/	/	/	**/**	/	/	/	**/**	1	/	4.76 (1.59, 14.27)	**.005**	/	/	/	**/**
≤14	5 (5)	0.0%	1.62 (1.34, 1.97)	**<.001**	3	0.0%	2.62 (1.69, 4.05)	**<.001**	2	.0%	3.52 (1.63, 7.60)	**.001**	4	0.0%	2.80 (1.80, 4.36)	**<.001**
**Country**																
Europe	5 (5)	0.0%	1.71 (1.37, 2.14)	**<.001**	3	0.0%	2.96 (1.73, 5.08)	**<.001**	5	.0%	3.12 (2.00, 4.86)	**.030**	3	0.0%	**2.67 (1.70, 4.20)**	**<.001**
Asia	6 (5)	74.3%	2.44 (1.49, 4.02)	**<.001**	2	20.4%	2.50 (1.53, 4.10)	**<.001**	1	.0%	2.34 (1.12, 9.38)	**<.001**	1	**/**	**7.42 (0.95, 57.96)**	.056
**Number of patients**																
≤100	8 (7)	67.2%	2.31 (1.57, 2.40)	**<.001**	3	0.0%	3.03 (1.83, 4.99)	**<.001**	6	.0%	3.14 (2.08, 4.73)	**<.001**	3	0.0%	2.67 (1.56, 4.57)	**<.001**
>100	3 (3)	0.0%	1.66 (1.29, 2.13)	**<.001**	2	2.9%	2.38 (1.40, 4.05)	**.001**	/	/	/	/	1	**/**	3.10 (1.43, 6.73)	**.004**
** Age**																
≤75	2 (2)	89.8%	2.84 (0.87, 9.29)	.085	3	0.0%	2.62 (1.69, 4.05)	**<.001**	2	.0%	3.06 (1.55, 6.03)	**.001**	2	**15.3%**	**2.51 (1.33, 6.74)**	**.004**
>75	9 (8)	38.6%	1.87 (1.46, 2.39)	**<.001**	2	0.0%	2.91 (1.50, 5.63)	**.002**	4	.0%	3.18 (1.90, 5.32)	**<.001**	1	0.0%	3.10 (1.43, 6.73)	**.004**
**Type of therapy**																
Chemotherapy	8 (8)	65.2%	2.07 (1.53, 2.78)	**<.001**	**/**	**/**	**/**	**/**	1	.0%	3.86 (1.27, 11.76)	**.017**	**/**	**/**	**/**	**/**
Immune checkpoint inhibitor	**/**	**/**	**/**	**/**	1	0.0%	1.99 (0.69, 5.75)	.204	3	.0%	2.68 (1.56, 4.59)	**<.001**	**/**	**/**	**/**	**/**
Radiotherapy	1 (1)	/	2.98 (1.34, 6.62)	**.007**	1	0.0%	2.04 (1.11, 3.75)	**.022**	2	.0%	3.90 (1.82, 8.38)	**<.001**	**/**	**/**	**/**	**/**

Bold values indicate statistically significant results (p < 0.05). Abbreviations: *N*, number of comparisons (studies); OS, overall survival; HR, Hazard Ratio; CI, Confidence interval.

### Publication bias and sensitivity analysis

A funnel plot was used to analyze the publication bias of the G-8 score in predicting OS and PFS. There was publication bias for OS and PFS according to the funnel plots (Figures S1A and S2B). Begg’s and Egger’s tests also showed a high probability of publication bias for OS (Egger’s test, *P* <.001; Begg’s test, *P* <.001). Sensitivity analyses were performed using the leave-one-out approach for OS (Figure S2A) and PFS (Figure S2B). The pooled results remained robust after the removal of individual studies.

## Discussion

This meta-analysis and systematic review aimed to evaluate the impact of frailty, as defined by G-8 scores, on cancer prognosis. The findings revealed a high prevalence of frailty among cancer patients, with variations depending on the type of tumor. The results demonstrated that patients with low G-8 sores had more advanced stages and higher PS scores. Furthermore, low G-8 scores were significantly associated with poor PFS and OS in cancer patients. Our meta-analysis also verified the predictive value of G-8 scores for survival in patients with cancer receiving nonsurgical treatment. The results also revealed that low G-8 scores predicted poor OS in patients with specific cancers.

As a complex, multidimensional, and cyclical state characterized by diminished physiological reserve, conceptually different from but distinctly related to aging, comorbidity, and disability, frailty is difficult to evaluate in a clinical setting.^73,74^ A tool that can rapidly and accurately screen patients with cancer for frailty is needed to identify patients who require CGA and make clinical decisions. The G-8 tool was developed by Soubeyran et al. in 2012 to rapidly identify frailty in elderly cancer patients who would benefit from CGA.^26^ As a screening tool, some articles reported that the G-8 and CGA were highly consistent in evaluating frailty. One study found no statistical difference between G-8 and CGA in diagnosing frailty in patients with breast cancer.^31^ The development of the G-8 tool was based on the CGA framework. By extracting the most relevant core indicators of CGA (such as nutrition, functional status, and cognition), the 2 indicators were found to be highly consistent in key dimensions to ensure consistency between the screening results and CGA results.

Notably, our findings demonstrate that G-8 scores are associated with poor survival in patients with cancer. The reason the G-8 tool can predict the prognosis of cancer patients can be explained by the following points: First, frailty is closely associated with cancer prognosis.^75^ The patients with frailty usually had a reduced physiological reserve. These patients had poor resistance to chemotherapy^76^ and were less likely to receive an optimal dose of chemotherapy.^77^ Among patients receiving radiotherapy, nonfrail patients had a higher capacity to receive concomitant and adjuvant chemotherapy.^78^ Additionally, preoperative frailty increased the risk of adverse discharge disposition and postoperative complications.^79^ Frailty was associated with a 2.95-fold increase in postoperative mortality.^80^ Owing to the inclusion of scores for reduced food intake and weight loss, the G-8 score reflects the nutritional status of patients to a certain extent. Numerous studies have confirmed that the prognosis of cancer patients with malnutrition is significantly worsened.^81,82^ Undernourished cancer patients tend to have a higher rate of discontinuation of anticancer therapy, higher rate of treatment toxicity, and significantly reduced survival time. In addition, cancer patients with malnutrition are more likely to experience cancer cachexia, which is a predictor of poor prognosis.^83,84^ Furthermore, studies have suggested that sarcopenia is an important risk factor for frailty.^85^ Patients with low G-8 scores were more likely to be diagnosed with sarcopenia.^86^ Sarcopenia had also been verified to be associated with poor OS and PFS and low rates of complete response and treatment completion in patients with cancer.^60,87^ Daily medication scores directly reflected the possibility of other comorbidities, and the general condition of patients with more comorbidities was worse.^88^ Psychological problems also directly affect patient tolerance to disease and treatment.^89^ Therefore, we posit that the G-8 tool serves as a promising and simple instrument for assessing and screening frailty in cancer patients, with its scoring system directly correlating to the survival of patients with cancer.

In clinical practice, ECOG performance status (ECOG-PS) is commonly used to roughly estimate the general condition of patients and is a reliable and consistent determinant of prognosis in several major tumor types and among patients of all ages.^90^ However, ECOG-PS assesses activities of daily living and does not consider age, comorbidities, or other aspects of frailty. ECOG-PS is a performance status measure, focusing on functional ability and daily activities. Frailty is broader, encompassing more than just physical function. Considering the great impact of frailty on the prognosis of cancer, we need to evaluate frailty status after ECOG-PS is performed. Our results indicated that G-8 scores were associated with ECOG-PS scores. Nevertheless, whether the G-8 or ECOG-PS scores are superior in predicting the prognosis of cancer patients and in diagnosing frailty remains to be further substantiated through additional research.

Additionally, many studies have found that patients with low G-8 scores have higher rates of advanced treatment-related toxicities.^51,55^ These results confirm that frailty has a comprehensive impact on the prognosis of cancer patients. Our subgroup study also found no significant association between a low G-8 score and short PFS in patients with lung cancer, and this association was also not significant in the subgroup with a cutoff value of 11 or in the radiotherapy subgroup. The main reason for this may be that very few studies were included.

Moreover, given that both cancer and anticancer therapies offered significant additional stressors that challenge the patients’ physiological reserves, the incidence of frailty in older patients with cancer was especially high.^91^ In previous meta-analysis, the results demonstrated that median prevalence of frailty was 42% (range 6%-86%).^11^ In our meta-analysis, the median prevalence of frailty across trials was 65.4% (range 27%-91%), which is consistent with previous studies. These results suggest that additional considerations should be taken when formulating anticancer treatment plans for cancer patients with frailty, and more attention should be paid to the patients’ treatment responses during the therapeutic process to manage symptoms promptly.

Our study had some limitations. Of the included studies, 19 were retrospective studies, 22 were prospective studies, and 1 was a case-control study. Some of the literature quality was not high, which affected the stability and robustness of the meta-analysis results. Second, the included studies included a variety of different tumor types and treatment modalities, which directly contributed to the heterogeneity among the articles. Third, the cutoff values of the G-8 score used in the included studies also varied in the meta-analysis.

## Conclusions

The prevalence of frailty is high among cancer patients. Decreased G-8 scores are significantly associated with poor survival in patients with cancer. The G-8 tool is a promising tool for screening frailty and predicting prognosis in patients with cancer.

## Supplementary Material

oyaf118_suppl_Supplementary_Tables_S1_Figures_S1-S2

## Data Availability

The datasets used and/or analyzed during the current study are available from the corresponding author on reasonable request.

## References

[1] Kiri S , Ryba T. Cancer, metastasis, and the epigenome. Mol Cancer. 2024;23:154. https://doi.org/10.1186/s12943-024-02069-w 39095874 PMC11295362

[2] Bray F , Laversanne M, Sung H, et al Global cancer statistics 2022: globocan estimates of incidence and mortality worldwide for 36 cancers in 185 countries. CA Cancer J Clin. 2024;74:229-263. https://doi.org/10.3322/caac.21834 38572751

[3] Han B , Zheng R, Zeng H, et al Cancer incidence and mortality in china, 2022. J Natl Cancer Cent. 2024;4:47-53. https://doi.org/10.1016/j.jncc.2024.01.006 39036382 PMC11256708

[4] Matsuda T , Saika K. Age-specific cancer incidence rate in the world. Jpn J Clin Oncol. 2020;50:626-627. https://doi.org/10.1093/jjco/hyaa057 32346738

[5] Pawelec G. Does patient age influence anti-cancer immunity? Semin Immunopathol. 2019;41:125-131. https://doi.org/10.1007/s00281-018-0697-6 30006738

[6] Feliu J , Heredia-Soto V, Girones R, et al Management of the toxicity of chemotherapy and targeted therapies in elderly cancer patients. Clin Transl Oncol. 2020;22:457-467. https://doi.org/10.1007/s12094-019-02167-y 31240462

[7] Komici K , Bencivenga L, Navani N, et al Frailty in patients with lung cancer: a systematic review and meta-analysis. Chest. 2022;162:485-497. https://doi.org/10.1016/j.chest.2022.02.027 35217002

[8] Clegg A , Young J, Iliffe S, Rikkert MO, Rockwood K. Frailty in elderly people. Lancet. 2013;381:752-762. https://doi.org/10.1016/S0140-6736(12)62167-9 23395245 PMC4098658

[9] Collard RM , Boter H, Schoevers RA, Oude Voshaar RC. Prevalence of frailty in community-dwelling older persons: a systematic review. J Am Geriatr Soc. 2012;60:1487-1492. https://doi.org/10.1111/j.1532-5415.2012.04054.x 22881367

[10] Ness KK , Wogksch MD. Frailty and aging in cancer survivors. Transl Res. 2020;221:65-82. https://doi.org/10.1016/j.trsl.2020.03.013 32360946 PMC7321876

[11] Handforth C , Clegg A, Young C, et al The prevalence and outcomes of frailty in older cancer patients: a systematic review. Ann Oncol. 2015;26:1091-1101. https://doi.org/10.1093/annonc/mdu540 25403592

[12] Guida JL , Hyun G, Belsky DW, et al Associations of seven measures of biological age acceleration with frailty and all-cause mortality among adult survivors of childhood cancer in the st. Jude lifetime cohort. Nat Cancer. 2024;5:731-741. https://doi.org/10.1038/s43018-024-00745-w 38553617 PMC11139608

[13] Patel I , Winer A. Assessing frailty in gastrointestinal cancer: two diseases in one? Curr Oncol Rep. 2024;26:90-102. https://doi.org/10.1007/s11912-023-01483-5 38180691

[14] Muhandiramge J , Orchard SG, Warner ET, van Londen GJ, Zalcberg JR. Functional decline in the cancer patient: a review. Cancers (Basel). 2022;14:1368. https://doi.org/10.3390/cancers14061368 35326520 PMC8946657

[15] Jespersen E , Winther SB, Minet LR, Möller S, Pfeiffer P. Frailty screening for predicting rapid functional decline, rapid progressive disease, and shorter overall survival in older patients with gastrointestinal cancer receiving palliative chemotherapy - a prospective, clinical study. J Geriatr Oncol. 2021;12:578-584. https://doi.org/10.1016/j.jgo.2020.10.007 33830020

[16] Cao X , Yang Z, Li X, et al Association of frailty with the incidence risk of cardiovascular disease and type 2 diabetes mellitus in long-term cancer survivors: a prospective cohort study. BMC Med. 2023;21:74. https://doi.org/10.1186/s12916-023-02774-1 36829175 PMC9951842

[17] Mohile SG , Dale W, Somerfield MR, et al Practical assessment and management of vulnerabilities in older patients receiving chemotherapy: Asco guideline for geriatric oncology. J Clin Oncol. 2018;36:2326-2347. https://doi.org/10.1200/JCO.2018.78.8687 29782209 PMC6063790

[18] Wildiers H , Heeren P, Puts M, et al International society of geriatric oncology consensus on geriatric assessment in older patients with cancer. J Clin Oncol. 2014;32:2595-2603. https://doi.org/10.1200/JCO.2013.54.8347 25071125 PMC4876338

[19] Parker SG , McCue P, Phelps K, et al What is comprehensive geriatric assessment (cga)? An umbrella review. Age Ageing. 2018;47:149-155. https://doi.org/10.1093/ageing/afx166 29206906

[20] Welsh TJ , Gordon AL, Gladman JR. Comprehensive geriatric assessment--a guide for the non-specialist. Int J Clin Pract. 2014;68:290-293. https://doi.org/10.1111/ijcp.12313 24118661 PMC4282277

[21] Ellis G , Gardner M, Tsiachristas A, et al Comprehensive geriatric assessment for older adults admitted to hospital. Cochrane Database Syst Rev. 2017;9:CD006211. https://doi.org/10.1002/14651858.CD006211.pub3 28898390 PMC6484374

[22] Lundqvist M , Alwin J, Henriksson M, et al Cost-effectiveness of comprehensive geriatric assessment at an ambulatory geriatric unit based on the age-fit trial. BMC Geriatr. 2018;18:32. https://doi.org/10.1186/s12877-017-0703-1 29386007 PMC5793378

[23] Oncology ISoG. 2025. Available at https://siog.org/resources/resources-siog/comprehensive-geriatric-assessment/

[24] Goede V. Frailty and cancer: current perspectives on assessment and monitoring. Clin Interv Aging. 2023;18:505-521. https://doi.org/10.2147/CIA.S365494 37013130 PMC10066705

[25] Rowbottom L , Loucks A, Jin R, et al Performance of the vulnerable elders survey 13 screening tool in identifying cancer treatment modification after geriatric assessment in pre-treatment patients: a retrospective analysis. J Geriatr Oncol. 2019;10:229-234. https://doi.org/10.1016/j.jgo.2018.10.018 30420323

[26] Bellera CA , Rainfray M, Mathoulin-Pelissier S, et al Screening older cancer patients: first evaluation of the g-8 geriatric screening tool. Ann Oncol. 2012;23:2166-2172. https://doi.org/10.1093/annonc/mdr587 22250183

[27] Garcia MV , Agar MR, Soo WK, To T, Phillips JL. Screening tools for identifying older adults with cancer who may benefit from a geriatric assessment: a systematic review. JAMA Oncol. 2021;7:616-627. https://doi.org/10.1001/jamaoncol.2020.6736 33443547

[28] Pearce J , Swinson D, Cairns D, et al; GO2 trial investigators. Frailty and treatment outcome in advanced gastro-oesophageal cancer: an exploratory analysis of the go2 trial. J Geriatr Oncol. 2022;13:287-293. https://doi.org/10.1016/j.jgo.2021.12.009 34955446 PMC8986151

[29] Morimoto K , Yamada T, Takeda T, et al Prospective observational study evaluating the prognostic value of the g8 screening tool for extensive-stage small cell lung cancer patients who received programmed death-ligand 1 inhibitor plus platinum-etoposide chemotherapy. Drugs Aging. 2023;40:563-571. https://doi.org/10.1007/s40266-023-01034-4 37145245

[30] Sekiguchi S , Tsuchiya K, Yasui Y, et al Clinical usefulness of geriatric assessment in elderly patients with unresectable hepatocellular carcinoma receiving sorafenib or lenvatinib therapy. Cancer Rep (Hoboken). 2022;5:e1613. https://doi.org/10.1002/cnr2.1613 35302279 PMC9675392

[31] Scheepers ERM , van der Molen LF, van den Bos F, et al The g8 frailty screening tool and the decision-making process in older breast cancer patients. Eur J Cancer Care (Engl). 2021;30:e13357. https://doi.org/10.1111/ecc.13357 33159382

[32] Yajima S , Masuda H. The significance of g8 and other geriatric assessments in urologic cancer management: a comprehensive review. Int J Urol. 2024;31:607-615. https://doi.org/10.1111/iju.15432 38402450

[33] van Walree IC , Scheepers E, van Huis-Tanja L, et al A systematic review on the association of the g8 with geriatric assessment, prognosis and course of treatment in older patients with cancer. J Geriatr Oncol 2019;10:847-858. https://doi.org/10.1016/j.jgo.2019.04.016 31078444

[34] Horiuchi K , Kuno T, Takagi H, Egorova NN, Afezolli D. Predictive value of the g8 screening tool for postoperative complications in older adults undergoing cancer surgery: a systematic review and meta-analysis. J Geriatr Oncol. 2024;15:101656. https://doi.org/10.1016/j.jgo.2023.101656 37940482

[35] Pearce J , Martin S, Heritage S, et al Frailty and outcomes in adults undergoing systemic anti-cancer treatment: a systematic review and meta-analysis. J Natl Cancer Inst. 2025;Jan 30:djaf017. https://doi.org/10.1093/jnci/djaf01 39886936 PMC12232047

[36] Agemi Y , Shimokawa T, Sasaki J, et al Prospective evaluation of the g8 screening tool for prognostication of survival in elderly patients with lung cancer: a single-institution study. PLoS One. 2019;14:e0210499. https://doi.org/10.1371/journal.pone.0210499 30653558 PMC6336333

[37] Anic K , Altehoefer C, Krajnak S, et al The preoperative g8 geriatric screening tool independently predicts survival in older patients with endometrial cancer: results of a retrospective single-institution cohort study. J Cancer Res Clin Oncol. 2023;149:851-863. https://doi.org/10.1007/s00432-022-03934-1 35212815 PMC9931812

[38] Anic K , Birkert S, Schmidt MW, et al G-8 geriatric screening tool independently predicts progression-free survival in older ovarian cancer patients irrespective of maximal surgical effort: results of a retrospective cohort study. Gerontology. 2022;68:1101-1110. https://doi.org/10.1159/000520328 34875663

[39] Bai LY , Li CP, Shan YS, et al A prospective phase ii study of biweekly s-1, leucovorin and gemcitabine in elderly patients with locally advanced or metastatic pancreatic adenocarcinoma. J Clin Oncol. 2022;40:550-550. https://doi.org/10.1200/jco.2022.40.4_suppl.550 35932625

[40] Bakas AT , Polinder-Bos HA, Streng F, et al Frailty in non-geriatric patients with head and neck cancer. Otolaryngol Head Neck Surg. 2023;169:1215-1224. https://doi.org/10.1002/ohn.388 37264978

[41] Banna GL , Basso U, Giunta EF, et al The geriatric g8 score is associated with survival outcomes in older patients with advanced prostate cancer in the adhere prospective study of the meet-uro network. Curr Oncol. 2022;29:7745-7753. https://doi.org/10.3390/curroncol29100612 36290889 PMC9600362

[42] Beardo P , Osman I, San José B, et al Safety and outcomes of new generation hormone-therapy in elderly chemotherapy-naive metastatic castration-resistant prostate cancer patients in the real world. Arch Gerontol Geriatr. 2019;82:179-185. https://doi.org/10.1016/j.archger.2019.02.008 30818172

[43] Becherini C , Banini M, Desideri I, et al Clinical outcome of nivolumab in older and frail patients with recurrent/metastatic head and neck squamous cell carcinoma. Journal of Geriatric Oncology. 2023;14:101380. https://doi.org/10.1016/j.jgo.2022.09.009 36175350

[44] Bentsen KK , Hansen O, Ryg J, Giger AW, Jeppesen SS. Combination of the g-8 screening tool and hand-grip strength to predict long-term overall survival in non-small cell lung cancer patients undergoing stereotactic body radiotherapy. Cancers (Basel). 2021;13:3363. https://doi.org/10.3390/cancers13133363 34282772 PMC8269387

[45] Couderc AL , Tomasini P, Nouguerède E, et al Older patients treated for lung and thoracic cancers: unplanned hospitalizations and overall survival. Clin Lung Cancer. 2021;22:e405-e414. https://doi.org/10.1016/j.cllc.2020.06.004 32665168

[46] Deluche E , Leobon S, Lamarche F, Tubiana-Mathieu N. First validation of the g-8 geriatric screening tool in older patients with glioblastoma. J Geriatr Oncol. 2019;10:159-163. https://doi.org/10.1016/j.jgo.2018.07.002 30037767

[47] Fujita K , Lee S, Morishita T, et al Prognostic significance of the geriatric 8 score alone and included with genetic risk group in older adults with acute myeloid leukemia. J Geriatr Oncol. 2023;14:101582. https://doi.org/10.1016/j.jgo.2023.101582 37429106

[48] Gavriatopoulou M , Fotiou D, Roussou M, et al Vulnerability variables among octogenarians myeloma patients: a single-center analysis in 110 patients. Leuk Lymphoma. 2019;60(3):619-628. https://doi.org/10.1080/10428194 30628505

[49] Ishii R , Ogawa T, Ohkoshi A, et al Use of the geriatric-8 screening tool to predict prognosis and complications in older adults with head and neck cancer: a prospective, observational study. J Geriatr Oncol. 2021;12:1039-1043. https://doi.org/10.1016/j.jgo.2021.03.008 33757718

[50] Kadokura M , Mori Y, Takenaka Y, et al Usefulness of the g8 geriatric assessment tool as a prognostic factor in gemcitabine plus nab-paclitaxel combination therapy for elderly patients with pancreatic cancer. JMA J 2022;5:512-519. https://doi.org/10.31662/jmaj.2022-0086 36407075 PMC9646307

[51] Kobayshi K , Suyama K, Katsuya H, et al; KSCC1701 study group. A phase ii multicenter trial assessing the efficacy and safety of first-line s-1 + ramucirumab in elderly patients with advanced/recurrent gastric cancer: Kscc1701. Eur J Cancer. 2022;166:279-286. https://doi.org/10.1016/j.ejca.2022.02.028 35349925

[52] Krenzlin H , Jankovic D, Alberter C, et al Frailty in glioblastoma is independent from chronological age. Front Neurol. 2021;12:777120. https://doi.org/10.3389/fneur.2021.777120 34917020 PMC8669893

[53] Kubo T , Watanabe H, Ninomiya K, et al Immune checkpoint inhibitor efficacy and safety in older non-small cell lung cancer patients. Jpn J Clin Oncol. 2020;50:1447-1453. https://doi.org/10.1093/jjco/hyaa152 32869100

[54] Lee S , Fujita K, Morishita T, et al Association of the geriatric 8 with treatment intensity and prognosis in older patients with diffuse large b-cell lymphoma. Br J Haematol. 2021;194:325-335. https://doi.org/10.1111/bjh.17554 34041751

[55] Liao Z , Zhao L, Xiong X, et al Impaired geriatric 8 score is associated with worse survival after radical intensity modulated radiation therapy in elderly patients with nasopharyngeal carcinoma. Acta Otolaryngol. 2023;143:334-339. https://doi.org/10.1080/00016489.2023.2190769 36994877

[56] Liposits G , Ryg J, Skuladottir H, et al Prognostic value of baseline functional status measures and geriatric screening in vulnerable older patients with metastatic colorectal cancer receiving palliative chemotherapy – the randomized nordic9-study. J Geriatr Oncol. 2023;14:101408. https://doi.org/10.1016/j.jgo.2022.11.007 36494261

[57] Loi M , Comito T, Franzese C, et al Charlson comorbidity index and g8 in older old adult(≥80 years) hepatocellular carcinoma patients treated with stereotactic body radiotherapy. J Geriatr Oncol. 2021;12:1100-1103. https://doi.org/10.1016/j.jgo.2021.01.001 33461945

[58] Maebayashi T , Ishibashi N, Aizawa T, et al Significance of stereotactic body radiotherapy in older patients with early stage non-small cell lung cancer. J Geriatr Oncol. 2018;9:594-599. https://doi.org/10.1016/j.jgo.2018.03.005 29573969

[59] Martinez-Tapia C , Paillaud E, Liuu E, et al; ELCAPA Study Group. Prognostic value of the g8 and modified-g8 screening tools for multidimensional health problems in older patients with cancer. Eur J Cancer. 2017;83:211-219. https://doi.org/10.1016/j.ejca.2017.06.027 28750273

[60] Middelburg JG , Middelburg RA, van Zwienen M, et al; LPRO (Dutch National Organization for Radiotherapy in the Elderly). Impaired geriatric 8 score is associated with worse survival after radiotherapy in older patients with cancer. Clin Oncol (R Coll Radiol). 2021;33:e203-e210. https://doi.org/10.1016/j.clon.2020.09.002 32972801

[61] Momota M , Hatakeyama S, Soma O, et al Geriatric 8 screening of frailty in patients with prostate cancer. Int J Urol. 2020;27:642-648. https://doi.org/10.1111/iju.14256 32500621

[62] Nakazawa J , Kawahira M, Kawahira M, et al Analysis of factors affecting progression-free survival of first-line chemotherapy in older patients with advanced gastrointestinal cancer. J Geriatr Oncol. 2021;12:1200-1207. https://doi.org/10.1016/j.jgo.2021.05.006 33994149

[63] Pottel L , Lycke M, Boterberg T, et al G-8 indicates overall and quality-adjusted survival in older head and neck cancer patients treated with curative radiochemotherapy. BMC Cancer. 2015;15:875. https://doi.org/10.1186/s12885-015-1800-1 26553007 PMC4640221

[64] Rao AR , Noronha V, Ramaswamy A, et al Assessing frailty in older indian patients before cancer treatment: comparative analysis of three scales and their implications for overall survival. J Geriatr Oncol. 2024;15:101736. https://doi.org/10.1016/j.jgo.2024.101736 38428186

[65] Rier HN , Meinardi MC, van Rosmalen J, et al Association between geriatric assessment and post-chemotherapy functional status in older patients with cancer. Oncologist. 2022;27:e878-e888. https://doi.org/10.1093/oncolo/oyac131 35861263 PMC9632320

[66] Shah M , Noronha V, Ramaswamy A, et al G8 and ves-13 as screening tools for geriatric assessment and predictors of survival in older indian patients with cancer. J Geriatr Oncol. 2022;13:720-730. https://doi.org/10.1016/j.jgo.2022.02.013 35283049

[67] Shiotsu S , Yoshimura A, Yamada T, et al Pembrolizumab monotherapy for untreated pd-l1-positive non-small cell lung cancer in the elderly or those with poor performance status: a prospective observational study. Front Oncol. 2022;12:904644. https://doi.org/10.3389/fonc.2022.904644 36158655 PMC9504658

[68] Soubeyran P , Bellera C, Goyard J, et al Screening for vulnerability in older cancer patients: the oncodage prospective multicenter cohort study. PLoS One. 2014;9:e115060. https://doi.org/10.1371/journal.pone.0115060 25503576 PMC4263738

[69] Takahashi M , Sakamoto Y, Ohori H, et al Phase ii study of trifluridine/tipiracil (tas-102) therapy in elderly patients with colorectal cancer (t-core1401): geriatric assessment tools and plasma drug concentrations as possible predictive biomarkers. Cancer Chemother Pharmacol. 2021;88:393-402. https://doi.org/10.1007/s00280-021-04277-3 34028598 PMC8316169

[70] Takahashi M , Takahashi M, Komine K, et al The g8 screening tool enhances prognostic value to ecog performance status in elderly cancer patients: a retrospective, single institutional study. PLoS One. 2017;12:e0179694. https://doi.org/10.1371/journal.pone.0179694 28640844 PMC5480957

[71] Wilk M , Waśko-Grabowska A, Skoneczna I, Szczylik C, Szmit S. Cardiac biomarkers and geriatric assessment in metastatic castrate-resistant prostate cancer during abiraterone acetate therapy - a cardio-oncology study. Cancer Control. 2022;29:10732748221140696. https://doi.org/10.1177/10732748221140696 36447439 PMC9716601

[72] Zurlo IV , Pozzo C, Strippoli A, et al Safety and efficacy of a first-line chemotherapy tailored by g8 score in elderly metastatic or locally advanced gastric and gastro-esophageal cancer patients: a real-world analysis. Geriatrics (Basel). 2022;7:107. https://doi.org/10.3390/geriatrics7050107 36286210 PMC9601931

[73] Rodriguez-Manas L , Feart C, Mann G, et al; FOD-CC group (Appendix 1). Searching for an operational definition of frailty: a delphi method based consensus statement: the frailty operative definition-consensus conference project. J Gerontol A Biol Sci Med Sci. 2013;68:62-67. https://doi.org/10.1093/gerona/gls119 22511289 PMC3598366

[74] Fried LP , Ferrucci L, Darer J, Williamson JD, Anderson G. Untangling the concepts of disability, frailty, and comorbidity: implications for improved targeting and care. J Gerontol A Biol Sci Med Sci. 2004;59:255-263. https://doi.org/10.1093/gerona/59.3.m255 15031310

[75] Alamgeer M , Ling RR, Ueno R, et al Frailty and long-term survival among patients in australian intensive care units with metastatic cancer (frail-cancer study): a retrospective registry-based cohort study. Lancet Healthy Longev. 2023;4:e675-e684. https://doi.org/10.1016/S2666-7568(23)00209-X 38042160

[76] Runzer-Colmenares FM , Urrunaga-PastorD, Roca-MoscosoMA, et alFrailty and vulnerability as predictors of chemotherapy toxicity in older adults: a longitudinal study in peru. J Nutr Health Aging. 2020;24:966-972. https://doi.org/10.1007/s12603-020-1404-633155622

[77] Narasimhulu DM , McGree ME, Weaver AL, et al Frailty is a determinant of suboptimal chemotherapy in women with advanced ovarian cancer. Gynecol Oncol. 2020;158:646-652. https://doi.org/10.1016/j.ygyno.2020.05.046 32518016

[78] Guzeloz Z , Gorken IB, Aydin B, et al Evaluation of treatment outcomes and tolerability in older patients with rectal cancer treated with radiotherapy accompanied by the g-8 geriatric score: Trod13-003 multicenter study. J Geriatr Oncol. 2024;15:101739. https://doi.org/10.1016/j.jgo.2024.101739 38492350

[79] Shaw JF , Budiansky D, Sharif F, McIsaac DI. The association of frailty with outcomes after cancer surgery: a systematic review and metaanalysis. Ann Surg Oncol. 2022;29:4690-4704. https://doi.org/10.1245/s10434-021-11321-2 35072860

[80] Davey MG , Joyce WP. Impact of frailty on oncological outcomes in patients undergoing surgery for colorectal cancer - a systematic review and meta-analysis. Surgeon. 2023;21:173-180. https://doi.org/10.1016/j.surge.2022.06.001 35792005

[81] Bullock AF , Greenley SL, McKenzie GAG, Paton LW, Johnson MJ. Relationship between markers of malnutrition and clinical outcomes in older adults with cancer: systematic review, narrative synthesis and meta-analysis. Eur J Clin Nutr. 2020;74:1519-1535. https://doi.org/10.1038/s41430-020-0629-0 32366995 PMC7606134

[82] Fu J , Xu X, Tian M, Wang H, Jin X. The controlling nutritional status score as a new prognostic predictor in patients with cervical cancer receiving radiotherapy: a propensity score matching analysis. BMC Cancer. 2024;24:1093. https://doi.org/10.1186/s12885-024-12872-9 39227776 PMC11370220

[83] Baracos VE , Martin L, Korc M, Guttridge DC, Fearon KCH. Cancer-associated cachexia. Nat Rev Dis Primers. 2018;4:17105. https://doi.org/10.1038/nrdp.2017.105 29345251

[84] Xu X , Tian M, Ding CC, et al Skeletal muscle index-based cachexia index as a predictor of prognosis in patients with cancer: a meta-analysis and systematic review. Nutr Rev. 2025;83:e852-e865. https://doi.org/10.1093/nutrit/nuae094 39001797

[85] Mori H , Tokuda Y. Differences and overlap between sarcopenia and physical frailty in older community-dwelling japanese. Asia Pac J Clin Nutr. 2019;28:157-165. https://doi.org/10.6133/apjcn.201903_28(1).0021 30896427

[86] Cavusoglu C , Tahtaci G, Dogrul RT, et al Predictive ability of the g8 screening test to determine probable sarcopenia and abnormal comprehensive geriatric assessment in older patients with solid malignancies. BMC Geriatr. 2021;21:574. https://doi.org/10.1186/s12877-021-02544-9 34666690 PMC8524815

[87] Xu XT , He DL, Tian MX, Wu H-J, Jin X. Prognostic value of sarcopenia in patients with diffuse large b-cell lymphoma treated with r-chop: a systematic review and meta-analysis. Front Nutr. 2022;9:816883. https://doi.org/10.3389/fnut.2022.816883 35284466 PMC8914205

[88] Sarfati D , Koczwara B, Jackson C. The impact of comorbidity on cancer and its treatment. CA Cancer J Clin. 2016;66:337-350. https://doi.org/10.3322/caac.21342 26891458

[89] Panigrahi G , Ambs S. How comorbidities shape cancer biology and survival. Trends Cancer. 2021;7:488-495. https://doi.org/10.1016/j.trecan.2020.12.010 33446449 PMC8137526

[90] Blagden SP , Charman SC, Sharples LD, Magee LRA, Gilligan D. Performance status score: do patients and their oncologists agree? Br J Cancer. 2003;89:1022-1027. https://doi.org/10.1038/sj.bjc.6601231 12966419 PMC2376959

[91] Mohile SG , Xian Y, Dale W, et al Association of a cancer diagnosis with vulnerability and frailty in older medicare beneficiaries. J Natl Cancer Inst. 2009;101:1206-1215. https://doi.org/10.1093/jnci/djp239 19638506 PMC3937788

