# Supplementary material for: The prognostic role of Geriatric 8 in patients with cancer: a meta-analysis and systematic review
Source: Oncologist. 2025 Jun 21;30(6):oyaf118. doi: 10.1093/oncolo/oyaf118 (PMC12204759; doi:10.1093/oncolo/oyaf118)

Supplementary files

The prognostic role of Geriatric 8 in patients with cancer: a meta-analysis and systematic review

Table S1. Risk of bias of the trials included in the meta-analysis

| Study | year | Country | Study  Participation | Study  Attrition | Prognostic Factor  Measurement | Outcome  Measurement | Study  Confounding | Statistical Analysis  and Reporting | Overall Risk  of Bias |
| --- | --- | --- | --- | --- | --- | --- | --- | --- | --- |
| Lies Pottel | 2015 | Belgium | L | L | L | L | H | L | L |
| Masahiro Takahashi | 2017 | Japan | M | L | L | L | M | L | M |
| Yoko Agemi | 2019 | Japan | H | L | L | L | L | L | L |
| Elise Deluche | 2019 | France | M | L | L | L | M | L | M |
| Toshio Kubo | 2020 | Japan | M | L | L | L | L | L | L |
| Masaki Momota | 2020 | Japan | M | L | L | L | M | H | M |
| Kristian Kirkelund Bentsen | 2021 | Denmark | L | L | L | L | M | L | L |
| Anne-Laure Couderc | 2020 | France | L | L | L | L | L | L | L |
| Katharina Anic | 2023 | Germany | M | M | L | L | L | L | M |
| Li-Yuan Bai | 2022 | China | L | L | L | L | H | M | M |
| Ajay T. Bakas | 2023 | Netherlands | H | L | L | L | H | L | H |
| Giuseppe Luigi Banna | 2022 | Italy | L | L | L | L | L | L | L |
| Carlotta Becherini | 2023 | Italy | M | L | L | L | H | L | M |
| Kazuma Kobayshi | 2022 | Japan | L | M | L | L | H | L | M |
| Ryo Ishii | 2021 | Japan | M | L | L | L | M | L | M |
| Eva Jespersen | 2021 | Denmark | L | L | L | L | L | L | L |
| Shin Lee | 2021 | Japan | M | L | L | L | L | L | L |
| Mauro Loi | 2021 | Italy | M | L | L | L | H | M | M |
| Ellen R. M. Scheepers | 2020 | Netherlands | M | L | L | L | H | L | M |
| Pastora Beardo | 2019 | Spain | M | L | L | L | M | H | M |
| Kei Fujita | 2023 | Japan | M | L | L | L | L | L | L |
| Maria Gavriatopoulou | 2019 | Greece | L | L | L | L | M | L | L |
| Kenji Morimoto | 2023 | Japan | L | L | L | L | L | L | L |
| Makoto Kadokura | 2022 | Japan | M | L | L | L | L | L | L |
| Harald Krenzlin | 2021 | Germany | M | L | L | L | H | L | M |
| Zhaohui Liao | 2023 | China | M | L | L | L | L | L | L |
| Gabor Liposits | 2023 | Denmark | L | L | L | L | L | L | L |
| Ina Valeria Zurlo | 2022 | Italy | M | L | L | L | L | L | L |
| Michał Wilk | 2022 | Poland | L | L | L | L | H | L | L |
| Masanobu Takahashi | 2021 | Japan | L | L | L | L | H | L | L |
| Shinsuke Shiotsu | 2022 | Japan | L | H | L | L | H | L | H |
| Minit Shah | 2022 | India | M | L | L | L | H | L | M |
| Shuhei Sekiguchi | 2022 | Japan | M | L | L | L | L | L | L |
| Hánah N. Rier | 2022 | Netherlands | M | L | L | L | H | L | M |
| Jessica Pearce | 2022 | UK | M | L | L | L | M | L | M |
| Junichi Nakazawa | 2021 | Japan | L | L | L | L | M | L | L |
| Katharina Anic | 2022 | Germany | L | L | L | L | M | L | L |
| Toshiya Maebayashi | 2018 | Japan | H | L | L | L | L | L | L |
| Claudia Martinez-Tapia | 2017 | France | L | M | L | L | L | L | L |
| J.G. Middelburg | 2020 | Netherlands | L | M | L | L | L | L | L |
| Abhijith R. Rao | 2024 | India | M | M | L | L | L | L | M |
| Pierre Soubeyran | 2014 | France | L | H | L | L | L | L | L |

L: low risk; M: moderate risk; H: high risk


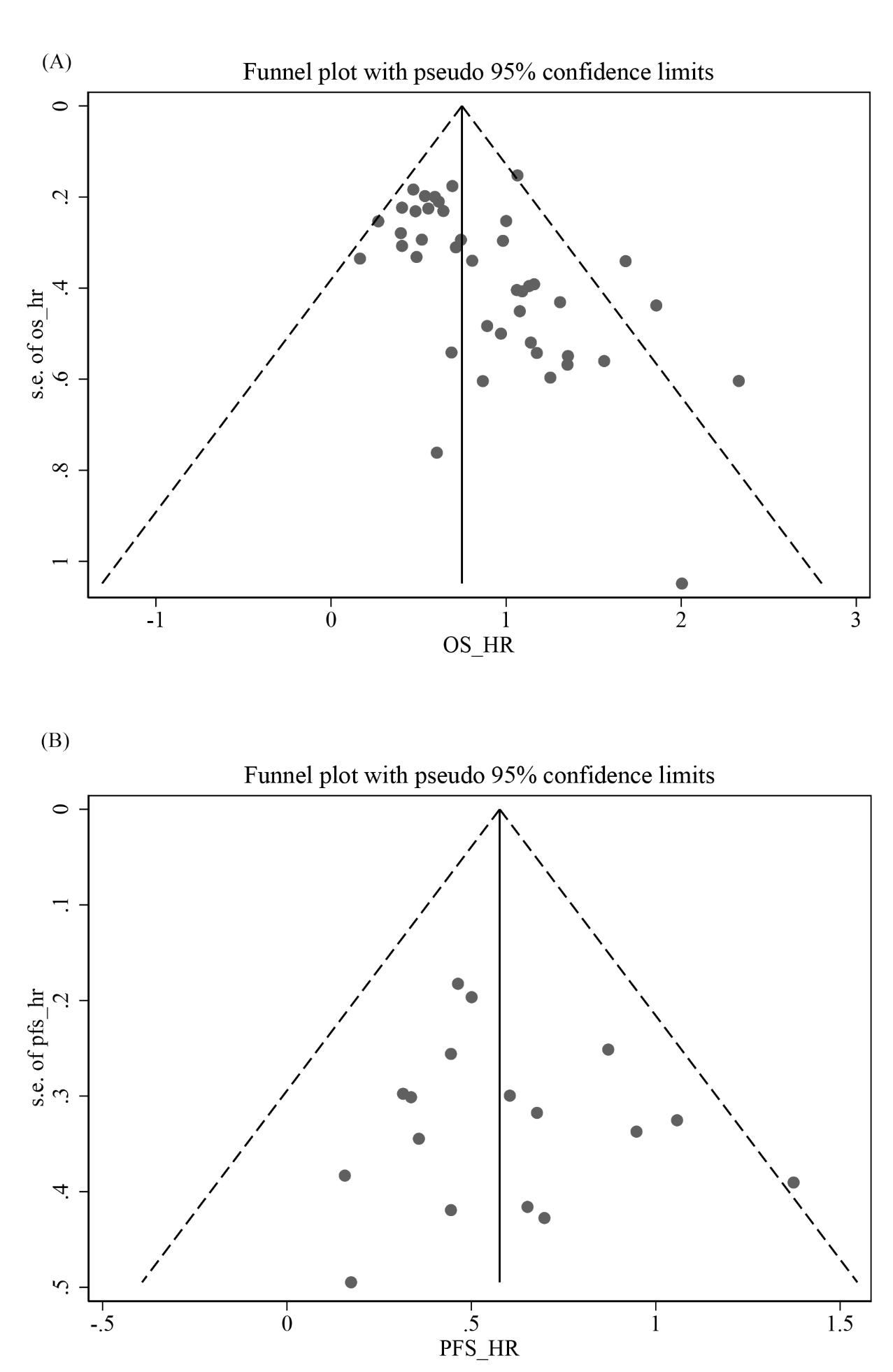
Figure S1 Publication bias analysis of meta-analysis on survival. (A) overall survival. (B) progression-free survival.

Figure S2 Sensitivity analysis of meta-analysis on survival. (A) overall survival. (B) progression-free survival.


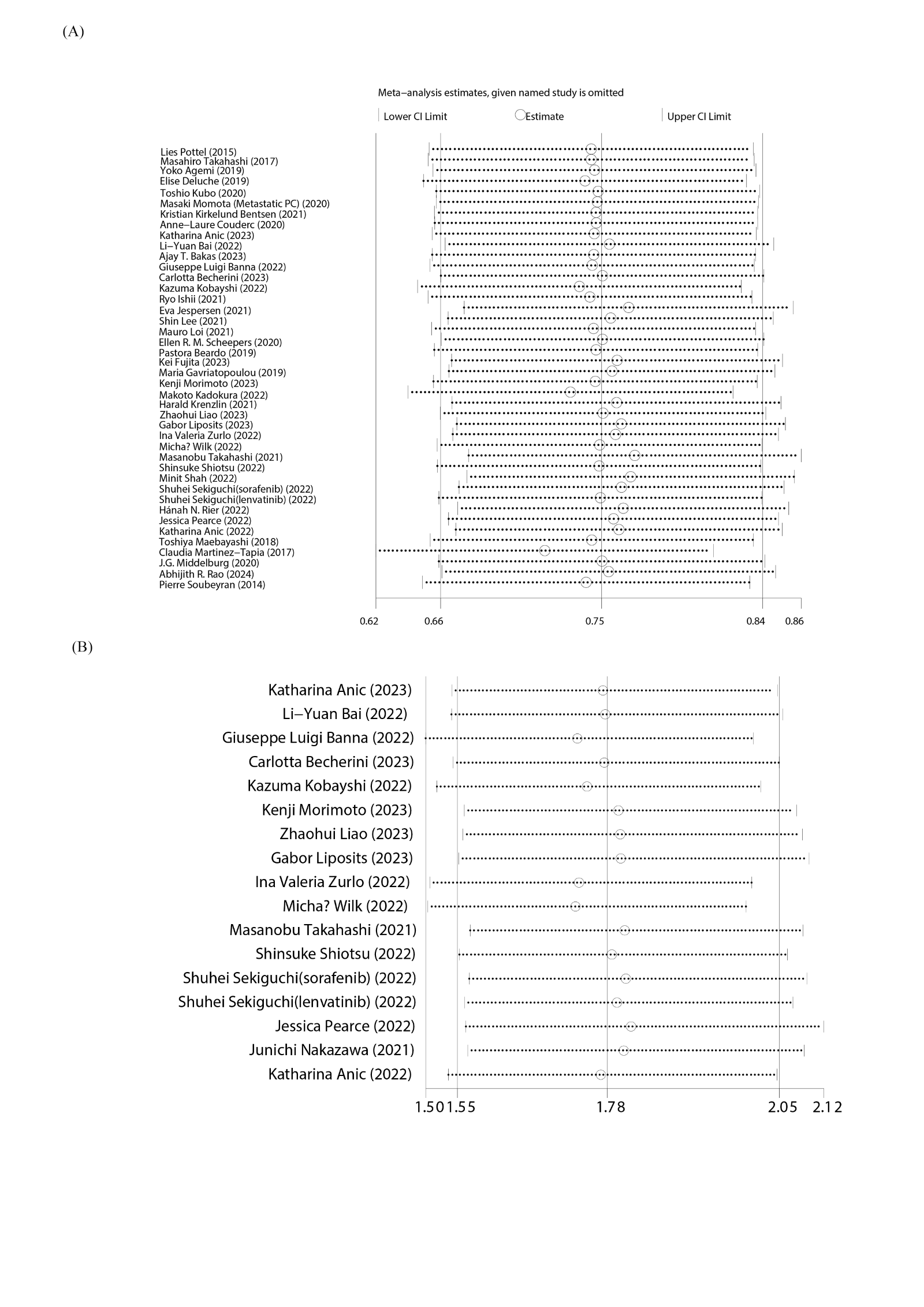

Supplement: oyaf118_suppl_Supplementary_Tables_S1_Figures_S1-S2 [file oyaf118_suppl_supplementary_tables_s1_figures_s1-s2.docx]
